# Contrasting effect of hybridization on genetic differentiation in three rockfish species with similar life history

**DOI:** 10.1111/eva.13749

**Published:** 2024-07-19

**Authors:** Anita Wray, Eleni Petrou, Krista M. Nichols, Robert Pacunski, Larry LeClair, Kelly S. Andrews, Marty Kardos, Lorenz Hauser

**Affiliations:** ^1^ School of Aquatic and Fishery Sciences University of Washington Seattle Washington USA; ^2^ Conservation Biology Division, Northwest Fisheries Science Center, National Marine Fisheries Service, NOAA Seattle Washington USA; ^3^ Washington Department of Fish and Wildlife Olympia Washington USA; ^4^ Zoology Department Nelson Mandela University Gqeberha South Africa; ^5^ Present address: United States Geological Survey, Alaska Science Center Anchorage Alaska USA

**Keywords:** fisheries management, hybridization, population genetics, *Sebastes auriculatus*, *Sebastes caurinus*, *Sebastes maliger*

## Abstract

Hybridization can provide evolutionary benefits (e.g., population resilience to climate change) through the introduction of adaptive alleles and increase of genetic diversity. Nevertheless, management strategies may be designed based only on the parental species within a hybrid zone, without considering the hybrids. This can lead to ineffective spatial management of species, which can directly harm population diversity and negatively impact food webs. Three species of rockfish (Brown Rockfish (*Sebastes caurinus*), Copper Rockfish (*S. auriculatus*), and Quillback Rockfish (*S. maliger*)) are known to hybridize within Puget Sound, Washington, but genetic data from these species are used to infer population structure in the entire genus, including in species that do not hybridize. The goal of this project was to estimate the hybridization rates within the region and determine the effect of hybridization on geographic patterns of genetic structure. We sequenced 290 Brown, Copper, and Quillback rockfish using restriction‐site associated DNA sequencing (RADseq) from four regions within and outside Puget Sound, Washington. We show that (i) hybridization within Puget Sound was asymmetrical, not recent, widespread among individuals, and relatively low level within the genome, (ii) hybridization affected population structure in Copper and Brown rockfish, but not in Quillback Rockfish and (iii) after taking hybridization into account we found limited directional dispersal in Brown and Copper rockfish, and evidence for two isolated populations in Quillback Rockfish. Our results suggest that rockfish population structure is species‐specific, dependent on the extent of hybridization, and cannot be inferred from one species to another despite similar life history.

## 1 INTRODUCTION

Hybridization between closely related species is known to play an important role in population structure and evolution (Arnold, 1992). In many species, natural hybridization occurs at the periphery of a species range (Pfennig et al., 2016), in areas with empty niches or low abundance (Yakimowski & Rieseberg, 2014), or where there is a biogeographical barrier (Hobbs et al., 2022). These hybrid hotspots are often regions where individuals of mixed ancestry can persist for multiple generations. Hybridization can have detrimental effects through outbreeding depression and genetic assimilation (Hails & Morley, 2005), but it can also be an important evolutionary process that increases overall genetic diversity (Hamilton & Miller, 2016), transfers adaptive alleles among species (Hedrick, 2013; Jones et al., 2018), and be instrumental in the evolution of species flocks (McCartney et al., 2003; Salzburger et al., 2002). Hybridization may also increase adaptive potential in the face of changing environments (Arnold et al., 2008; Hamilton & Miller, 2016). Hybrid individuals can provide a space to maintain genetic diversity, especially where parental species are at risk of extinction (Soulé, 1985).

Despite the evolutionary benefits of natural hybridization, management regarding hybrids remains uncertain (Allendorf et al., 2001). In the United States, there is no robust policy regarding the conservation of hybrids, and their protection is still assessed on a case‐by‐case basis. Additionally, spatial management of the parental species can become complex, as hybrid zones contain individuals with alleles from both (or multiple) parental species. Therefore, localized hybridization may inflate estimates of intraspecific genetic differentiation in the parental species and may lead to an underestimation of connectivity and inflate the number of distinct populations within a single species. Disentangling the relative contributions of hybridization and within‐species connectivity is critically important for the accurate identification of management units.

Most research regarding natural hybridization has been focused on terrestrial plants and animals and species where hybrids are easily distinguishable. Although originally thought to occur less often in the marine environment, hybrids have now been documented in a wide range of marine plant and animal taxa (Arnold & Fogarty, 2009). To date, there are over 111 examples of hybridization among 173 marine fish species (Montanari et al., 2016). Three instances of marine hybridization have been identified in rockfishes (genus *Sebastes*) (Muto et al., 2013; Roques et al., 2001; Seeb, 1998), a highly diverse genus of over 110 described species, 90 of which are found in the eastern Pacific Ocean (Love et al., 2002). Originating in the late Miocene, *Sebastes* underwent a rapid radiation event creating many sister and cryptic species (Hyde & Vetter, 2007). One well‐known example of hybridization within *Sebastes* was found in Puget Sound, Washington, between Copper, Brown, and Quillback rockfish (*S. caurinus*, *S. auriculatus*, and *S. maliger*) (Seeb, 1998). These three species have a similar distribution from Baja California to Kodiak Island, Alaska, although Brown Rockfish are rare along the Washington and Oregon coasts (Love et al., 2002). Their depth preferences are also similar, ranging from subtidal to 270 meters, although Quillback Rockfish descends to depths deeper than the other two species. They also share life‐history characteristics, such as ovoviviparity, high fecundity, pelagic larval duration (2.5–3 months), and long lifespans (34–95 years) (Love et al., 2002). Buonaccorsi et al. (2002, 2005) and Schwenke et al. (2018) confirmed that introgression, i.e., the persistent gene flow of one species into another through repeated backcrossing, occurs within the Copper‐Brown‐Quillback species complex. However, introgression appears to be asymmetric, from Quillback into both Brown and Copper rockfish (Schwenke et al., 2018). Additionally, this hybridization is not recent as there is little evidence of early‐generation hybrids from studies using 18 allozyme (Seeb, 1998), six microsatellite (Buonaccorsi et al., 2002, 2005), or three nuclear and one mitochondrial loci (Schwenke et al., 2018). As these studies identified hybrids from a relatively small number of loci, the extent of introgression, frequency of hybrids, and timing of introgression remain poorly understood.

In addition to detecting hybridization among Puget Sound rockfishes, previous studies also found evidence of within‐species population structure; specifically, Brown, Copper, and Quillback rockfish from the Puget Sound estuary were found to be genetically distinct from populations on the Pacific coast of Washington State (Buonaccorsi et al., 2002, 2005; Seeb, 1998). Based in part on these studies, two distinct population segments (DPS) within the Puget Sound or Georgia Basin ecosystem and one along the Washington Coast were identified for each of the three species (Stout et al., 2001). These genetic findings were highly influential in deciding whether other rockfish species in Puget Sound could be considered independent DPSs, including three species that were subsequently listed under the U.S. federal Endangered Species Act as threatened or endangered (Drake et al., 2010). Subsequent genetic analysis confirmed significant population structure and the existence of a DPS for Yelloweye Rockfish (*S. ruberrimus*), but also revealed a lack of population structure for Canary Rockfish (*S. pinniger*, Andrews et al., 2018). These results informed a subsequent decision to remove Canary Rockfish from the endangered species list (NMFS, 2017). The lack of population structure in Canary Rockfish raised the possibility that the number of distinct spatial management units was overestimated in some other species. In the case of Copper/Brown/Quillback rockfish, intraspecific genetic population structure could be inflated by localized hybridization.

The objective of this study was to investigate whether interspecific hybridization and introgression had a significant effect on metrics of genetic population structure for Brown, Copper, and Quillback rockfish in Puget Sound. Specifically, we (1) quantified the extent of hybridization between species throughout Puget Sound, (2) estimated within‐species population structure and connectivity to identify distinct population segments, and (3) evaluated the effect of hybridization on population structure.

## 2 METHODS

### 2.1 Sampling procedure

**FIGURE 1:**
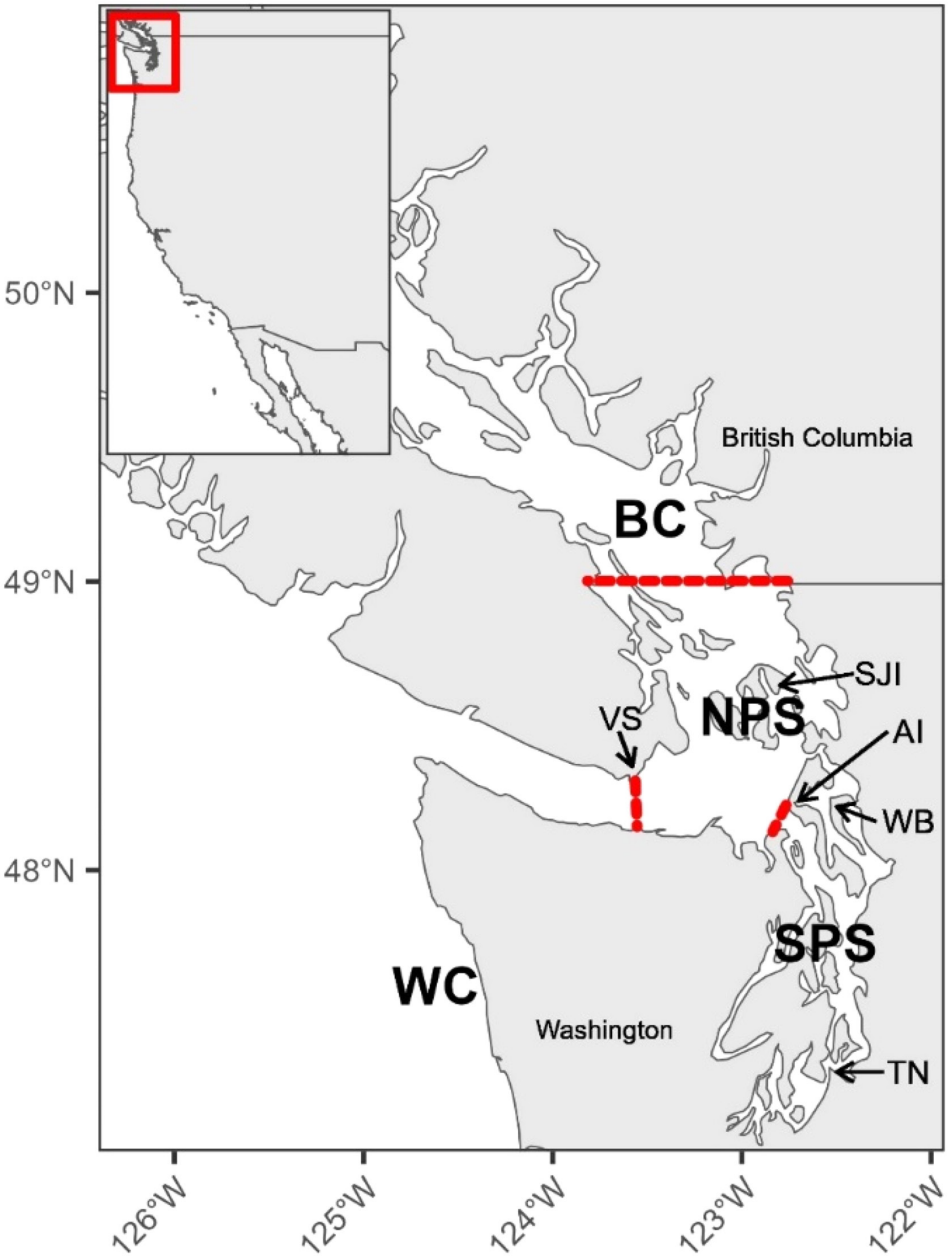
Brown, Copper, and Quillback Rockfish collection regions. Sampling areas within the Salish Sea are North Puget Sound (NPS), South Puget Sound (SPS) and British Columbia (BC). Red dashed lines show the Victoria sill (left), Admiralty Inlet (right), and the US/Canada border (top, as defined by this study) which separate our four regions. AI, Admiralty Inlet; SJI, San Juan Islands; TN, Tacoma Narrows; VS, Victoria Sill; WB, Whidbey Basin; Brown coastal individuals are from southern California and Mexico due to sample availability & species distribution.

We used 290 individuals from three species of rockfish (Brown, Copper, and Quillback) that were previously collected by the Washington Department of Fish and Wildlife (WDFW), the National Marine Fisheries Service Northwest Fisheries Science Center (NWFSC), and the Department of Fisheries and Oceans (DFO Canada) from 1999 to 2021 (Figure 1, Table 3). Tissue was clipped from individual fins and either preserved in 95% ethanol or dried. Individuals were collected from four regions: (1) British Columbia (Canadian Salish Sea north of US/Canada border, BC); (2) northern Puget Sound (US Salish Sea north of Admiralty Inlet, south of the US/Canada border and east of the Victoria sill, NPS); (3) southern Puget Sound (Puget Sound proper, south of Admiralty Inlet, SPS); and (4) the US West Coast (US Pacific Coast west of Victoria sill, WC) (Figure 1). We also re‐sampled 62 individuals that were identified as ‘pure’ parental species in a previous study, including 26 Brown, eight Copper, and 28 Quillback rockfish (Schwenke et al., 2018, detailed in Table S1). Due to differences in the abundance and distribution of species across this geographic range, Brown Rockfish could only be sampled in three of these locations and the WC individuals were collected in Mexico and southern California.

### 2.2 DNA extraction, library preparation, and sequencing

Genomic DNA was extracted using the Nexttec DNA isolation kit (Nexttec Incorporated, Middlebury, VT, USA) following the manufacturer's protocol and quantified using a Qubit fluorometer (ThermoFisher Scientific, Waltham, MA, USA). DNA concentration was normalized to 125 ng in 10 μL of molecular‐grade water. Restriction site‐associated DNA sequencing (RADseq) libraries were prepared using a version of the Ali et al. (2016) protocol without the targeted bait capture step, referred to in the literature as BestRAD (https://github.com/merlab‐uw/Protocols/blob/main/bestRAD). Briefly, genomic DNA was digested using the *Sbf*I enzyme. An adapter (P1) containing a forward amplification primer site, an Illumina sequencing primer site, and an individual 6 bp barcode was ligated to each fragment at the restriction site end. Fragments were then randomly sheared using sonication and size‐selected to 300–500 bp in length. Subsequently, P2 adapters were ligated to the reverse end, and libraries were amplified by PCR. Each library was assessed for quality on a 1% agarose gel and a Bioanalyzer DNA 1000 kit (Agilent Technologies, Santa Clara, CA). Libraries were pooled in equimolar amounts and sequenced on a NovaSeq at the University of Oregon. Individual samples were sequenced at paired ends (300 cycles) on either a S4 or SP run type. Individuals were assigned to one of six RADseq libraries randomly to avoid any lane effect.

### 2.3 Initial filtering

Raw sequence data were quality checked using *FastQC* v0.11.9 (Andrews, 2010) and visualized in *MultiQC* (Ewels et al., 2016). Prior to SNP calling and genome alignment, the raw sequences were demultiplexed using *process_radtags* in *STACKS* v2.60 (Catchen et al., 2011; Rochette et al., 2019). Here, sequences were trimmed to 104 bases and filtered for quality. Individuals with fewer than 250,000 total reads were excluded from downstream analysis. Our paired‐end sequences were then aligned to the Honeycomb Rockfish (*S. umbrosus*) genome from GenBank (NCBI Accession Number: PRJNA562243) with *Bowtie* 2 v2.4 using the ‘*very‐sensitive*’ option (Langmead & Salzberg, 2012). At the time of data analysis for this study, the Honeycomb Rockfish genome was one of only two annotated full genomes and was chosen due to its closer phylogenetic relationship to our species (Hyde & Vetter, 2007). Following genome alignment, SNP calling and basic population genetics statistics were calculated using the *gstacks* (*marukilow* model) and *populations* modules from the *STACKS* pipeline. SNPs were called if they had a minimum mapping quality of 40. SNPs were filtered following published recommendations (O'Leary et al., 2018) requiring that loci meet the following criteria: minimum genotype depth ≥5, mean minimum read depth ≥ 15, and genotype call rate ≥ 80%.

### 2.4 Interspecific analyses

For the interspecific analyses, the first SNP on each RADtag was chosen using the *–write‐single‐snp* option in *populations*. Only one SNP per RADtag was chosen to minimize the potential for highly linked loci. We did not select diagnostic SNPs (*F*_ST_ = 1) because those loci would likely primarily distinguish between Brown Rockfish and the other two species, which are more closely related to each other than to Brown Rockfish (Hyde & Vetter, 2007). Additionally, we did not filter based on departure from Hardy‐Weinberg proportions (HWE), as introgression may cause a Wahlund effect (a reduction of heterozygosity due to population structure within a sample) that would influence *F*_IS_ and HWE *p*‐values. To visualize interspecific population structure patterns and identify individuals with mixed ancestry, we used principal components analyses (PCA) and *STRUCTURE* analysis, the two most commonly used approaches to describe population structure (Liu et al., 2020). First, to identify evidence of recent hybridization, we conducted PCA using the R package *adegenet* v2.1.8 (Jombart, 2008) and visualized the results using *ggplot2* v3.3.6 (Wickham, 2016). Second, we used *STRUCTURE* v2.3.4 to estimate admixture (Pritchard et al., 2000). Two replicates were run for *K* = 1–10 clusters with a burn‐in period of 10,000 iterations and 100,000 MCMC repetitions. *STRUCTURE* was run without a priori population knowledge and using the admixture model. We identified the range of likely *K* groups with *Structure Harvester* (Earl & vonHoldt, 2012), using the Δ*K* statistic (Evanno et al., 2005), and using the mean *L*(*K*). Lastly, pairwise and overall *F*_ST_ values (Weir & Cockerham, 1984) were estimated between all four sampling locations with 1000 bootstraps using the R package *hierfstat* v0.5–11 (Goudet, 2005). Values were considered statistically significant if the lower limit of the 95% bootstrap confidence interval did not overlap with zero.

*Fastsimcoal* (v2.8, Excoffier et al., 2021) was used to distinguish between patterns of hybridization and incomplete lineage sorting. Models with different admixture rates were compared, including (1) no gene flow, (2) unidirectional admixture (i.e., admixture from Quillback into Brown and Copper but not vice versa), and (3) bidirectional admixture (i.e., admixture between Quillback and Brown and Quillback and Copper) (Figure S1). For each model, *fastsimcoal* was run with 40 ECM optimization cycles and 150,000 coalescent simulations. This was repeated 100 times, with random starting parameter values. The best fit model was determined based on the lowest AIC value and highest likelihood. The distribution of the best likelihood values was calculated by running the model with the best parameter values 100 times. Mutation rate and generation time were taken from Kolora et al. (2021). The divergence time for the three species was taken from Hyde and Vetter (2007). SFS files were generated from the interspecific analysis using *easySFS* (Gutenkunst et al., 2009). We only included individuals from SPS in our models, as previous studies suggest they are more likely to hybridize in that region (Schwenke et al., 2018).

Admixture *f*_3_ statistics were calculated on the empirical data to assess whether intermediate individuals were due to admixture (Patterson et al., 2012). The R package *AdmixTools2* 2.0.0 (Maier et al., 2023) was used to test whether a population (target) is the result of admixture between two other source populations (source X and source Y). Individuals with suspected admixture (target) were identified from *STRUCTURE* analysis as having ≤95% ancestry from one species. These individuals were analyzed against individuals with ≥95% ancestry from either species (source populations). A negative *f*_3_ value and *Z* score provided evidence of admixture in the target population between the two parental and source populations.

### 2.5 Intraspecific analyses

For the intraspecific analyses, we retained one SNP on each RADtag with the highest minor allele frequency (MAF). Choosing the highest MAF SNP ensured that intraspecific datasets were different from the interspecific data so results could be compared and correlated to each other. Overall, 27%, 17%, and 32% of SNPs were shared between the interspecific data and the Brown, Copper, and Quillback intraspecific datasets, respectively. SNPs with genotype frequencies that deviated significantly from Hardy‐Weinberg Equilibrium (HWE) expectations were removed using the following procedure: *p* values were calculated across individuals for each population using an exact test based on Monte Carlo permutations of alleles within the R package *pegas* v1.1 (Paradis, 2010). *p* values were then combined for each locus using Fisher's combination of probabilities and adjusted to *q* values for the false discovery rate (Benjamini & Hochberg, 1995). Loci with *q* values below 0.05 were considered significantly out of HWE and removed from downstream analysis. For both the inter‐ and intraspecific datasets, genetic relatedness was calculated using an identity by descent estimate on all pairs of individuals within *PLINK* v1.07 (Purcell et al., 2007) to evaluate if our dataset included highly related individuals. *F*_IS_ was also calculated using *vcftools* v0.1.16 (Danecek et al., 2011). Final datasets were converted from vcf to other formats (GenePop and *STRUCTURE*) using *PGDSpider* v2.1.1.5 (Lischer & Excoffier, 2012).

We analyzed intraspecific population structure using the above three methods (PCA, *F*_ST_, and *STRUCTURE*). Additionally, we compared results from the *STRUCTURE* and PCA analyses with percent admixture ancestry to determine if there was a correlation between admixed ancestry and intraspecific clustering in both analyses. In the *STRUCTURE* analysis, we correlated percent admixture ancestry with each of the genetic groups in the intraspecific analysis. In the PCA, we correlated percent admixture ancestry with PC1. Finally, to rule out the effect of chromosomal inversions on population structure, we estimated linkage disequilibrium (*r* ^2^) within each chromosome using *PLINK* v1.07 (Purcell et al., 2007). *r* ^2^ values were then mapped on each chromosome to identify blocks of highly linked loci using the R function *LDheatmap* v1.0–6 (Shin et al., 2006). Chromosomes with loci in strong LD (*r* ^2^ > 0.5) over extended blocks (distance greater than 1 Mb) were analyzed separately using PCAs in *adegenet* v2.1.8 (Jombart, 2008) to determine whether individuals clustered in the three‐stripe patterns consistent with chromosomal inversions.

## 3 RESULTS

### 3.1 Interspecific analyses

We retained, on average, 11.7, 18, and 18.7 million reads per individual for Brown, Copper, and Quillback rockfish, respectively. From these reads, we retained 12,708 SNPs after filtering (from 67,009 SNPs identified) that were genotyped in more than 80% of all individuals across the three species (N_Quillback_ = 87; N_Brown_ = 37, N_Copper_ = 90). Twenty individuals differed in morphological and genetic species identification, suggesting misidentification in the field; they were thus removed from subsequent analyses. A PCA of the misidentified individuals suggested they were not hybrid individuals (Figure S2). *F*_ST_ values between species were large (*F*_ST_ = 0.4–0.7), statistically significant, and mirrored the phylogenetic relationship (Table S2).

**FIGURE 2:**
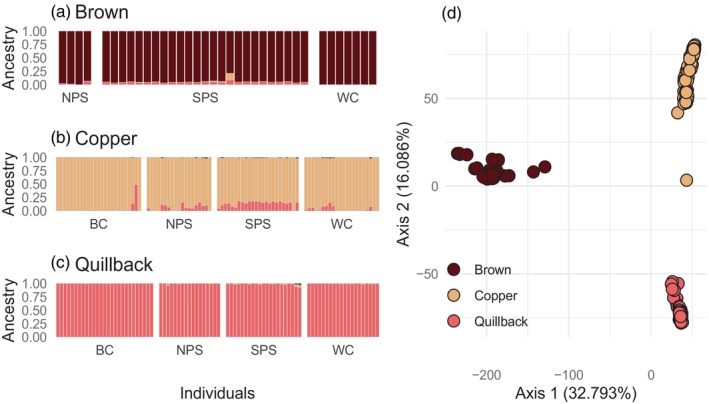
Interspecific *STRUCTURE* analysis (a–c) and PCA (d) of Brown, Copper and Quillback rockfish separated by geographic location. a–c: Each bar represents one sample, and the colors represent the ancestry proportions for each individual. The results are split by field identification: a = Brown Rockfish, b = Copper Rockfish, c = Quillback Rockfish. Individuals within each location are ordered south to north. d: Principal Components Analysis from all individuals suggests three clusters according to species. Individuals are color coded according to field identification. A clear F1 hybrid between Copper and Quillback is observed as intermediate along PC2.

The PCA showed three distinct clusters corresponding to the three species, with few intermediate individuals (Figure 2d). *STRUCTURE* suggested *K* = 2 (Figure S3) instead of the three species, which is likely due to the smaller genetic distance between Copper and Quillback compared to Brown Rockfish. We therefore present *K* = 3 which separated all three species. Admixture analysis with *STRUCTURE* revealed low levels of introgression from Quillback Rockfish into both Brown and Copper rockfish (Figure 2a–c). All Brown Rockfish individuals collected from SPS had low (~5%) Quillback ancestry. There were fewer Brown Rockfish with Quillback ancestry from NPS and none from the WC. Copper Rockfish had higher degrees of Quillback ancestry, with 92% of individuals in SPS showing >10% Quillback ancestry. Although less common, Copper Rockfish with admixed ancestry were also found in NPS (50% of individuals), WC (28% of individuals), and BC (8% of individuals). One Copper Rockfish individual from BC showed evidence of recent hybridization (48.8% Quillback and 51.2% Copper Rockfish ancestry, Figure 2b) and was an intermediate in the PCA (Figure 2d). We also identified three individuals with admixed ancestry (*N*_Brown_ = 1, *N*_Copper_ = 2) that were identified as ‘pure’ in a previous study (Schwenke et al., 2018). Admixed Quillback Rockfish were found only in SPS.

In coalescence simulations, the ‘bidirectional admixture’ model had by far the highest likelihood compared to the other models (Figure S4) and was most supported by model comparisons based on AIC. *F*_3_ statistics were highly and statistically significantly negative for both Copper‐Quillback and Brown‐Quillback admixed individuals (Table 1), confirming that source individuals were a result of admixture between two parental species.

**TABLE 1:** Admixture *f*_3_ statistics between admixed individuals and pure populations.

Target	Source X	Source Y	*f*_3_‐value	SE	*Z*‐score	*p*
Brown‐Admixed	Brown‐Pure	Quillback‐Pure	−0.00427	0.000176	−24.3	2.81E‐130
Copper‐Admixed	Copper‐Pure	Quillback‐Pure	−0.00204	0.000204	−10.0	1.05E‐23

*Note*: Values were calculated using the R package *AdmixTools* 2 2.0.0.

### 3.2 Intraspecific analyses

**FIGURE 3:**
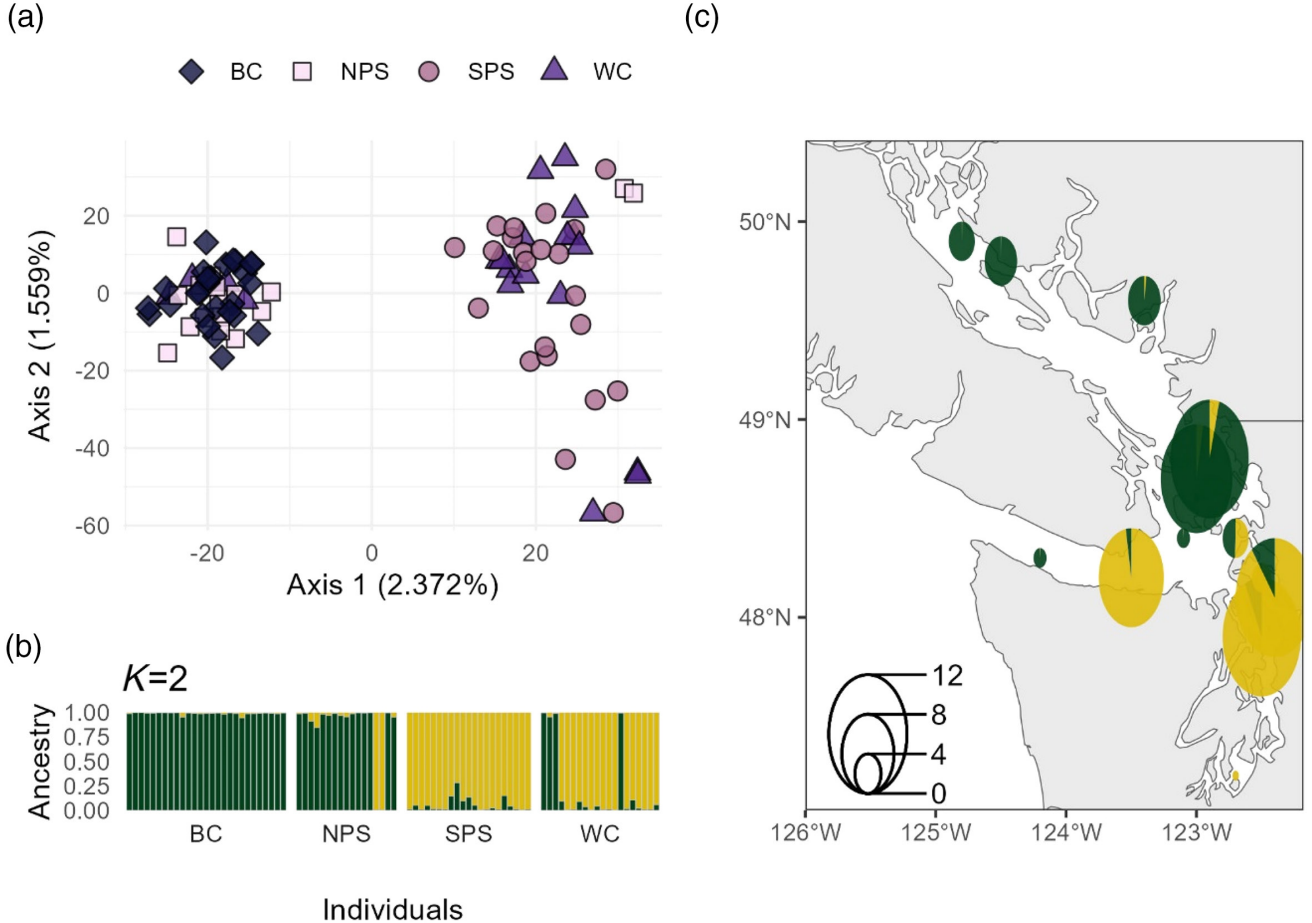
Intraspecific Principal Component Analysis and *STRUCTURE* plot for Quillback Rockfish. (a) Each point represents an individual fish, with color and shape representing the sampling location. (b) *STRUCTURE* plot for two groups, the most likely number of groups based on Δ*K*. Each bar represents an individual, and each color represents the proportion of the genome assigned to each genetic group. Individuals within each location are ordered south to north. (c) Geographic distribution of genetic groups. Pie charts are colored according to *STRUCTURE* plot results and adjusted for sample size (see legend in bottom left). The color of the pie corresponds to the average admixture proportions in each collection. Similar capture coordinates were pooled into the same pie.

When only Quillback Rockfish were used for SNP discovery, 607,854 SNPs were identified in *STACKS* and 8,525 SNPs were retained after filtering. We removed 20 individuals with low sequence read counts from our dataset. No Quillback Rockfish were removed due to misidentification. PCA revealed individuals sampled from SPS and WC formed one relatively large cluster, while individuals from NPS and BC formed a second, more tightly distributed cluster (Figure 3a). *STRUCTURE* suggested two genetic groups (*K* = 2, Figure S3), which followed the same patterns as the PCA (Figure 3b). Evidence for dispersal was present, as four individuals sampled from WC and two individuals from NPS were more genetically similar to the opposite group. Nevertheless, there was limited evidence for recent gene flow between populations as we identified few individuals with ancestry from both genetic groups (Figure 3b). *F*_ST_ values ranged from 0 for the SPS‐WC comparison to 0.013 for the SPS‐BC comparison (Table 2). Pairwise *F*_ST_ was statistically significantly greater than 0 for all pairwise comparisons between, but not within the two identified clusters.

**TABLE 2:** Pairwise Weir and Cockerham *F*_ST_ estimates for three species of rockfish in Puget Sound and the Washington Coast.

	Brown Rockfish				Copper Rockfish				Quillback Rockfish			
	SPS	NPS	BC	WC	SPS	NPS	BC	WC	SPS	NPS	BC	WC
SPS												
NPS	**0.049**				**0.049**				**0.009**			
BC					**0.09**	**0.019**			**0.013**	0.001		
WC	**0.181**	**0.105**			**0.057**	**0.002**	**0.014**		0	**0.006**	**0.009**	

*Note*: Bolded numbers are significantly greater than zero. Values were estimated with 1000 bootstraps using the R package *hierfstat* v0.5‐11 (Goudet, 2005).

**FIGURE 4:**
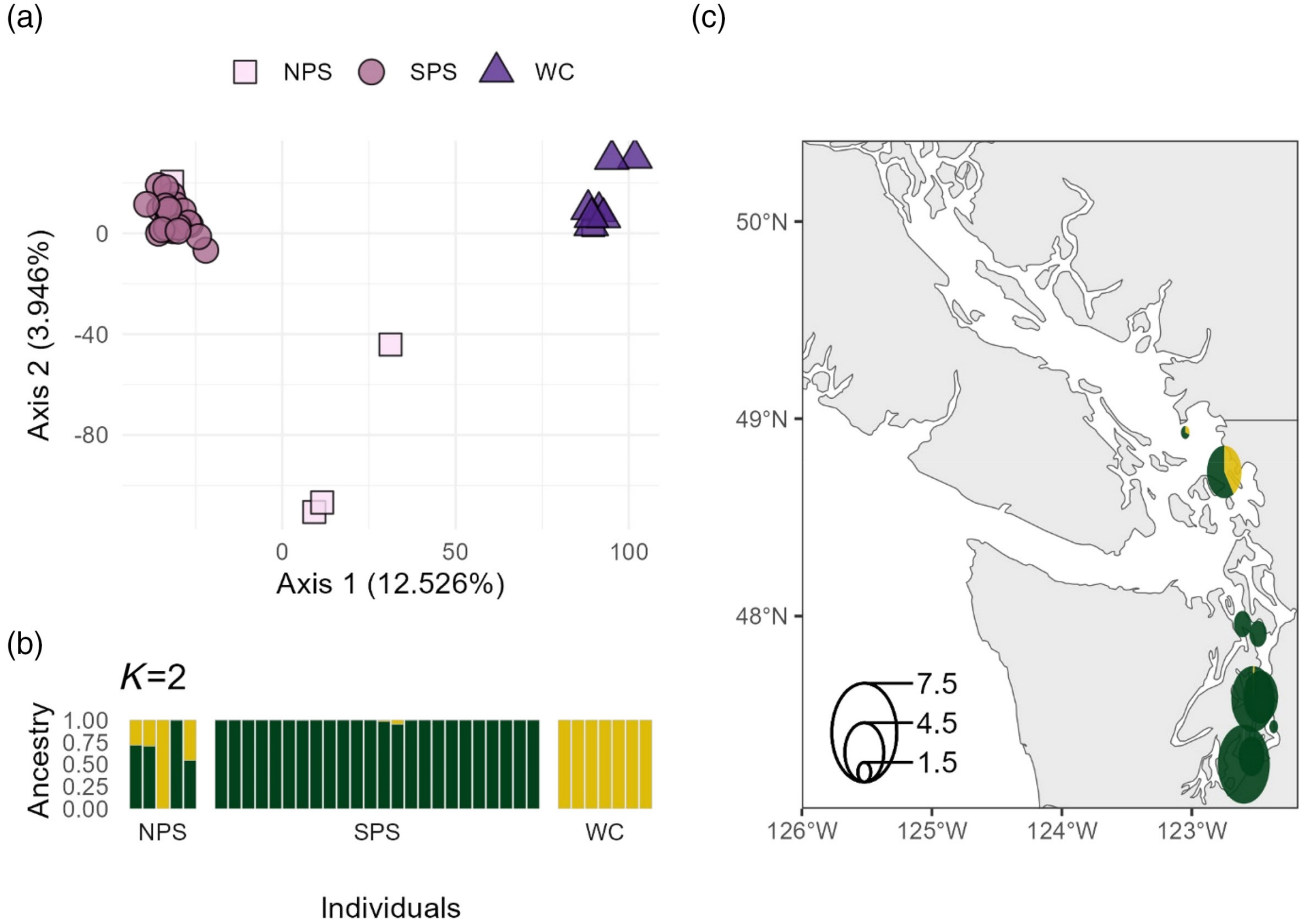
Intraspecific Principal Components Analysis and *STRUCTURE* plot for Brown Rockfish. (a) Each point represents an individual fish, with color and shape representing the sampling location. (b) *STRUCTURE* plot for two groups, the most likely number of groups based on Δ*K*. Each bar represents an individual, and each color represents the proportion of the genome assigned to each genetic cluster. Individuals within each location are ordered south to north. (c) Geographic distribution of genetic groups. Pie charts are colored according to *STRUCTURE* analyses and adjusted for sample size (see legend in bottom left). The color of the pie corresponds to the average admixture proportions in each collection. Similar capture coordinates were pooled into the same pie. Due to differences in the abundance and distribution of species across this geographic range, Brown Rockfish WC individuals were collected in Mexico and southern California and are not included in the map.

In Brown Rockfish, 279,876 SNPs were identified in *STACKS* and 10,055 SNPs were retained after filtering. We removed fourteen individuals from downstream analyses: eight because of low read count and six due to disagreement between genetic and morphological identification. Visualization of these individuals using a PCA in the interspecific analysis suggested that the six misidentified individuals were not hybrids but pure individuals of another species (Figure S2). Intraspecific PCA revealed two clusters separated along PC1 with some intermediate individuals from NPS that were separated along PC2 (Figure 4a). Correspondingly, *STRUCTURE* analyses suggested two genetic groups (*K* = 2, Figure 4b; Figure S3) primarily consisting of SPS and WC individuals, with NPS individuals having admixed ancestries between these two groups. The first PC of the PCA was highly correlated with the percent of Quillback admixture (*R* ^2^ = 0.84, Figure S5a). All pairwise *F*_ST_ values between locations were statistically significant (Table 2). Population‐based summary statistics (Table 3) identified a high *F*_IS_ value for NPS, possibly due to a Wahlund effect in a population mixture.

**TABLE 3:** Summary statistics for three species of rockfish in Puget Sound and the Washington Coast.

	BC	NPS	SPS	WC
Brown Rockfish				
*N*		5	23	8
*H*_O_		0.216	0.284	0.172
*H*_E_		0.236	0.280	0.165
*F*_IS_		−0.013	0.084	−0.040
Avg Admixed Ancestry		2.2%	4.9%	0%
Copper Rockfish				
*N*	23	18	23	20
*H*_O_	0.239	0.263	0.273	0.251
*H*_E_	0.246	0.279	0.0286	0.264
*F*_IS_	0.032	0.025	0.045	0.052
Avg Admixed Ancestry	2.6%	5.8%	13.7%	3.1%
Quillback Rockfish				
*N*	20	17	22	27
*H*_O_	0.252	0.271	0.279	0.266
*H*_E_	0.278	0.265	0.269	0.266
*F*_IS_	0.021	−0.021	−0.036	−0.002
Avg Admixed Ancestry	0%	0%	1%	1%

*Note*: All values were calculated using the R package *hierfstat* v0.5‐11 (Goudet, 2005). The average individual admixed ancestry was calculated using results from interspecific *STRUCTURE* analysis.

Abbreviations: *F*_IS_, inbreeding coefficient; *N*, number of individuals per sampling location; *H*_E_, average expected heterozygosity; *H*_O_, average observed heterozygosity.

**FIGURE 5:**
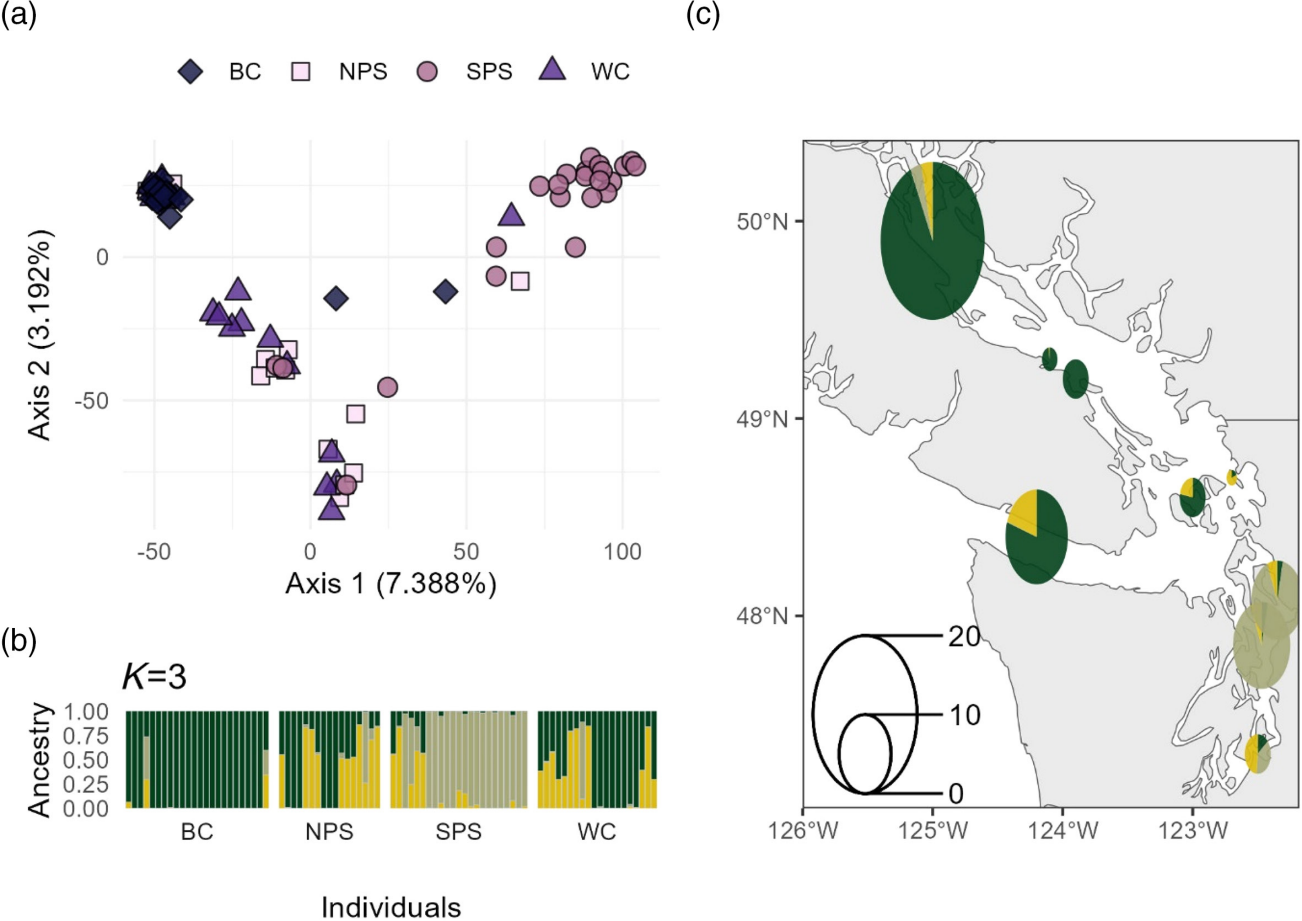
Intraspecific Principal Components Analysis and *STRUCTURE* Plot for Copper Rockfish. (a) Each point represents an individual fish, with color and shape representing the sampling location. (b) *STRUCTURE* plot for three groups, the most likely number of groups based on Δ*K*. Each bar represents an individual, and each color represents the proportion of the genome assigned to each genetic group. Individuals within each location are ordered south to north. (c) Geographic distribution of genetic groups. Pie charts are colored according to *STRUCTURE* analyses and adjusted for sample size (see legend in bottom left). The color of the pie corresponds to the average admixture proportions in each collection. Similar capture coordinates were pooled into the same pie.

In Copper Rockfish, 516,618 SNPs were identified in *STACKS* and 21,150 SNPs were retained after filtering. We removed 24 individuals from downstream analyses, 10 as a result of low read count and 14 as a result of disagreement between genetic and morphological identification. A PCA revealed that most individuals from BC and SPS formed two distinct groups along PC1, while individuals collected from all four locations were intermediate on PC1 and separated along PC2 (Figure 5a). *STRUCTURE* analyses suggested *K* = 3 (see Figure S6 for *K* = 4), with most of SPS and BC fish belonging to two distinct populations, while the remaining individuals were admixed between the BC population and a third group (Figure 5b). Pairwise *F*_ST_ values between locations were significantly >0 for all comparisons (Table 2), with the highest *F*_ST_ between BC and SPS.

**FIGURE 6:**
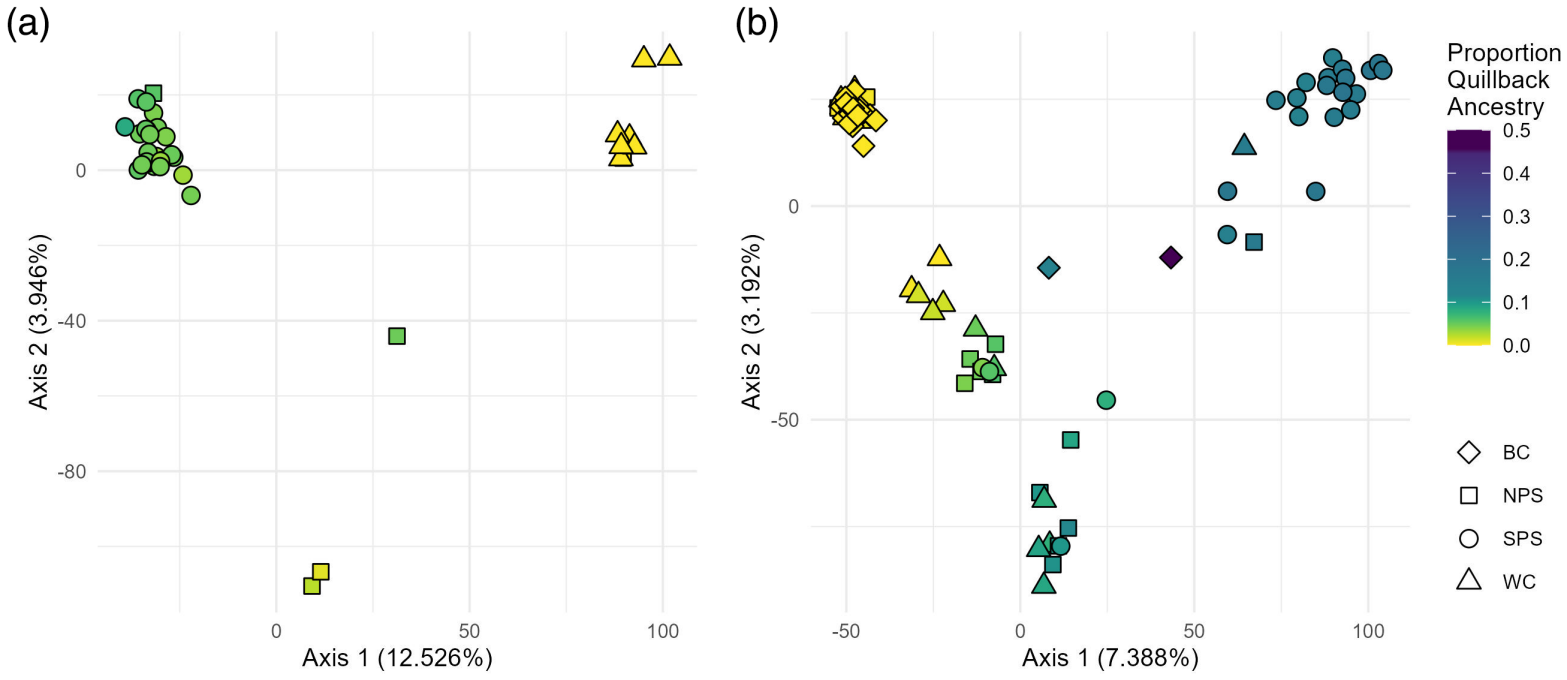
Hybridization rate strongly influences (a) Brown and (b) Copper intraspecific PCA analyses. Each circle represents an individual fish, colored by the percent Quillback ancestry. The shape defines the sampling location.

There was a strong correlation between inter‐ and intraspecific analyses in Copper Rockfish. Fish with a high proportion of Quillback ancestry (shown in pink in Figure 2b) had a low proportion of BC cluster ancestry (shown in dark green in Figure 5b) – this correlation was highly significant (*R* ^2^ = 0.67, Figure S5c). The percent Quillback ancestry was also strongly correlated with PC1 in the intraspecific analyses (*R* ^2^ = 0.71, Figure S5b and Figure 6b), suggesting that hybridization influences intraspecific genetic population differentiation.

### 3.3 Chromosomal inversion analyses

We identified a total of 23 blocks with elevated LD (eleven in Brown Rockfish, ten in Copper Rockfish, and two in Quillback Rockfish, Figure S7). No two species shared the same LD block. PCA of two LD blocks, one in Brown and one in Quillback, revealed a three‐banding pattern (Figure S8), consistent with chromosomal inversions, where the three bands show the three genotypes for the inversion. We identified no change in the PCA clustering patterns if SNPs within the inversions were incorporated into the analyses or not (data not shown). Additionally, we found no geographic patterns in inversion genotypes (Figure S8). We identified no LD blocks in Copper Rockfish that showed a three‐banding pattern.

## 4 DISCUSSION

Determining the extent of hybridization in Puget Sound rockfish is a key step towards understanding the influence of hybridization on genetic metrics of population structure and determination of management units (DPSs). This information is critical for the conservation and management of these species. The interspecific analysis suggested that (i) hybridization is low and asymmetric, (ii) admixed individuals largely remain in south Puget Sound, and (iii) hybridization is more common (i.e., more individuals were classified as having evidence of admixed ancestry), but the extent of admixture is lower than previously reported. The population structure was completely different for the three rockfish species. In Quillback Rockfish, there were two clusters in SPS/WC and BC/NPS, respectively, with almost no admixture between the clusters. In Brown Rockfish, we found two distinct clusters at the WC and in SPS, with admixed individuals in NPS. In Copper Rockfish, we found two distinct clusters in BC and SPS, respectively, with admixed individuals in all four regions. In both Copper and Brown rockfish, there was a strong correlation between the extent of Quillback introgression and population structure.

### 4.1 Interspecific hybridization

Our results and models provided evidence for widespread, low‐level, and asymmetric introgression of Quillback Rockfish into Brown and Copper rockfish. Although introgression is sometimes difficult to distinguish from incomplete lineage sorting (Holder et al., 2001), *f*_3_ statistics (Table 1) and *fastsimcoal* simulations (Figure S4) provided strong support for admixture. Furthermore, the geographic variation in admixture rates further supports hybridization as the main cause for the observed genetic patterns, as ancient shared polymorphisms from incomplete lineage sorting tend to be distributed randomly across species ranges rather than concentrated in specific hybrid zones (Wang et al., 2019). Although the three species co‐occur across most of their ranges, the hybrid zones in Puget Sound have been established independently from previous coast‐wide surveys with allozyme (Seeb, 1998) and microsatellite markers (Buonaccorsi et al., 2002, 2005), lending further support to the notion of introgression between species.

Although previous studies also reported asymmetric introgression, they identified more admixed Quillback individuals (Schwenke et al., 2018). The lower frequency of admixed Quillback individuals in our dataset could be due to our sampling locations, primarily in the north of the main basin of south Puget Sound. Using five mitochondrial and nuclear loci in Schwenke et al. (2018), Quillback individuals with admixed ancestry were identified south of the Tacoma Narrows (20% admixed individuals) and in the Whidbey Basin (19% admixed individuals). Seeb (1998) found admixed Quillback Rockfish in south Puget Sound, over 50 km south of our southernmost Puget Sound sampling site. Only one of our Quillback Rockfish individuals came from south of the Tacoma Narrows, and we purposefully selected ‘pure’ individuals as identified by Schwenke et al. (2018) from the Whidbey Basin. The absence of Quillback individuals with admixed ancestry in our dataset supports the finding that the proportion of Quillback hybridization increases with geographic isolation from the outer coast (Schwenke et al., 2018). Two distinct shallow sills separate Whidbey Basin and southern Puget Sound (Moore et al., 2008), which may retain hybrids within these isolated bays.

Consistent with Schwenke et al. (2018), we found higher admixture in Copper Rockfish than in Brown Rockfish. In contrast, prior microsatellite work detected extensive hybridization in Brown Rockfish (Buonaccorsi et al., 2005), but little evidence for introgressed alleles in Copper Rockfish (Buonaccorsi et al., 2002). However, microsatellites may not be able to identify introgressed individuals (Melville et al., 2017; Nielsen et al., 2014) because of extensive homoplasy between species (Henriques et al., 2016). For example, a study between two sister species of groupers (Genus *Plectropomus*) found diagnostic differentiation at one microsatellite locus but incomplete lineage sorting in another (van Herwerden et al., 2006). This discrepancy between loci could potentially drastically alter the degree of hybridization reported in a study.

The widespread but low level of Quillback introgression in Brown and Copper rockfish contrasts with previous reports. Given that temporal changes in genomic signatures of ancient hybridization between studies a few years apart are unlikely, this discrepancy may be due to the high genomic coverage of our RAD data allowing for more accurate estimates of hybridization. Hybrid identification depends on the number and type of markers used (Henriques et al., 2016), especially in systems with ancient introgression (Szatmári et al., 2021); generally, more markers allow the identification of more hybrids (McFarlane & Pemberton, 2019). Previous studies using few loci have observed a wide range in the proportion of admixed individuals. For example, 0% (Buonaccorsi et al., 2002) and 43% (Schwenke et al., 2018) of admixed Copper Rockfish individuals were found within the same geographic region (south of Admiralty Inlet, north of Tacoma Narrows), contrasting with the 96% found in this study. Using many more loci may also reduce the effect of differential introgression across the genome which may affect some loci more than others. Indeed, the higher admixture proportions in some Copper and Brown rockfish individuals reported in Schwenke et al. (2018) could be explained by adaptive introgression of the screened DNA markers. Introgression has long been identified as a source of adaptive genetic variation driving pesticide resistance in mice (Song et al., 2011) and beak traits in Darwin's finches (Grant & Grant, 2010). More research is needed to identify if adaptive introgression is occurring in Brown and Copper rockfish.

Our results add to the evidence that hybridization occurs primarily in south Puget Sound (Schwenke et al., 2018; Seeb, 1998, Table 3). Three primary potential hypotheses may account for this spatial pattern: habitat availability, anoxic conditions, and differences in population size (Schwenke et al., 2018). First, there is much less suitable rocky habitat in south Puget Sound compared to north Puget Sound, British Columbia, and outer coast waters (Miller & Borton, 1980), which may concentrate individuals, increasing spatial overlap between species and increasing hybridization potential. Second, Puget Sound experiences sporadic hypoxic conditions (Walt Deppe et al., 2013), which may force species into shallower habitats where oxygen concentrations are higher (Eby & Crowder, 2002). Since Quillback and Copper rockfish are typically separated by depth (Love et al., 2002), these anoxic periods may force Quillback Rockfish into Copper habitat, further increasing species overlap. These periods of hypoxia have occurred for centuries in Hood Canal, as indicated by sediment cores (Brandenberger et al., 2011), and they are consistent with the widespread and low‐level admixture observed in Brown and Copper rockfish which suggest historical hybridization events. Lastly, introgression may occur when a rare species fails to find suitable mates and thus hybridizes with a closely related common species (Hubbs, 1955). Quillback Rockfish are thought to be more abundant in Puget Sound than Brown and Copper rockfish, in both current and historical estimates (Schwenke et al., 2018). Periods of hypoxia can also cause severe rockfish die‐offs (Grantham et al., 2004), further eliminating available mates. This difference in population size suggests that the more common species, Quillback Rockfish, would hybridize with the less common species, Brown and Copper rockfish, leading to directional introgression.

### 4.2 Intraspecific population structure

Despite the similar ecology and life histories of Brown, Copper, and Quillback rockfish, patterns of population structure differed considerably. Our results show that interspecific hybridization affected intraspecific structure in Copper and Brown rockfish. Nevertheless, there was evidence for limited dispersal in all species, which can be used as a proxy for estimating population connectivity.

Quillback Rockfish consisted of two populations (BC/NPS and SPS/WC), with little interbreeding between populations. The north Puget Sound individuals in this study were collected further north near the San Juan Islands (Figure 3c), making connectivity between the West Coast and south Puget Sound without gene flow into north Puget Sound possible along the southern shores of the Strait of Juan de Fuca. These results suggest that the barrier for Quillback Rockfish would be the open water between Admiralty Inlet, the Victoria Sill, and the San Juan Islands. Shallow sills in fjord‐like marine environments are observed to act as a barrier to gene flow in Copper Rockfish along Vancouver Island (Dick et al., 2014), and Norwegian fjords for Atlantic Cod (*Gadus morhua*) (Knutsen et al., 2007), Blue Whiting (*Micromesistius poutassou*) (Giæver & Stien, 1998), and Pearlside (*Maurolicus muelleri*) (Suneetha & Nævdal, 2001), contributing to the evolution of isolated populations. Ocean circulatory features around Admiralty Inlet may therefore act as a barrier to larval dispersal (Khangaonkar et al., 2017; MacCready et al., 2021), in addition to limiting adult migration (Hannah & Rankin, 2011). Limited directional migration or dispersal out of the southern Puget Sound region for Quillback Rockfish follows similar results seen in Yelloweye Rockfish in Andrews et al. (2018). This finding in Quillback Rockfish could further justify the role of Admiralty Inlet in providing somewhat of a dispersal barrier for multiple rockfish species.

Our results from Brown Rockfish suggest that hybridization plays a large role in the substantial population genetic structure seen in this species. Additionally, our results provide evidence for significant population genetic differentiation between NPS, SPS, and southern California/Mexico, with similar *F*_ST_ values to a previous microsatellite study (Buonaccorsi et al., 2005). However, our study suggested that north Puget Sound may function as a mixing zone for coastal and south Puget Sound fish, as we found individuals with both SPS and southern California/Mexico ancestry in NPS (Figure 4c). This significant population structure may be attributable to the directional dispersal of admixed individuals from south Puget Sound. Similar mixing zone patterns have been seen in Turbot (*Scophthalmus maximus*) and Atlantic Cod (*Gadus morhua*) between the Atlantic Ocean/North Sea and Baltic Sea (Nielsen et al., 2003, 2004).

There are many hypotheses as to how mixing zones are maintained, including both intrinsic and extrinsic barriers such as geography, habitat unsuitability, and local adaptation (Nielsen et al., 2003, 2004). Adult Brown Rockfish have very small home ranges (~30 m^2^ in high‐relief artificial reefs, ~400 m^2^ in low‐relief habitat, Matthews, 1990), suggesting that any large‐scale movement occurs in the larval or juvenile stage. As previously mentioned, Admiralty Inlet is documented to restrict larval dispersal out of the south Puget Sound basin. After settlement, Brown Rockfish typically inhabit shallow water (< 30 meters), natural, and complex rocky habitats along Puget Sound proper (Stout et al., 2001). Due to the patchy habitat available in north Puget Sound (Palsson et al., 2009), it is possible that there is insufficient suitable habitat to support large population sizes for Brown, Copper, and Quillback rockfish. As a result, any available habitat in north Puget Sound is already filled with Copper and Quillback rockfish, which could suppress the population size of Brown Rockfish via interspecific competition.

Mixing zones may also be maintained by pre‐ or post‐zygotic barriers to reproduction. Brown Rockfish are hypothesized to have colonized Puget Sound after deglaciation of the region (Buonaccorsi et al., 2005), approximately 17,000 years ago (Mosher & Hewitt, 2004). The time since divergence between coastal and Puget Sound populations may have enabled local adaptation in geographically distinct populations of Brown Rockfish. Any interbreeding between two locally adapted populations may induce outbreeding depression (Allendorf et al., 2001), reinforcing the isolation of those two populations (Garant et al., 2007). However, empirical evidence for outbreeding depression in fish species seems limited (McClelland & Naish, 2007). In the case of Brown Rockfish, local adaptation and adaptive differentiation may be facilitated by introgressed Quillback alleles in south Puget Sound. Introgression has been documented to facilitate diversification and speciation in many animal species, including cichlids and cottids (Selz et al., 2014; Stemshorn et al., 2011). Finally, rockfish are documented to have complex courting rituals (Helvey, 1982) and apparent mate choice (Johansson et al., 2012). It is possible that preference for conspecific mates for coastal Brown Rockfish might promote divergence between coastal and Puget Sound populations, as seen in Swordtail (Willis et al., 2011). In north Puget Sound, where mate choice may be limited by small population sizes, choosiness might decrease and heterospecific (between hybrid and non‐hybrid individuals) mating could occur (Willis et al., 2011).

In Copper Rockfish, *STRUCTURE* identified three genetic clusters (*K* = 3). Two different genetic clusters were found in BC and SPS, respectively. All four regions had admixed individuals between the BC cluster and a third genetic group. The identity of that third genetic group is uncertain, as *STRUCTURE* did not identify any ‘pure’ individuals. The estimation of *K* is the most uncertain feature of *STRUCTURE* (and related programmes) (Novembre, 2016), which often cannot be reproduced even qualitatively and should be interpreted in a biological context (Gilbert et al., 2012). Although SPS and BC were therefore clearly alternative populations (one likely affected by hybridization), the population mixture in NPS and the WC may be an artifact, though this interpretation will have to be verified with additional samples from the West Coast.

Despite these issues with determining the true number of populations, Quillback ancestry was highly correlated with intraspecific genetic differentiation in both the PCA and *STRUCTURE* analyses. Thus, we cannot clearly disentangle patterns of hybridization with population structure for Copper Rockfish. Similar patterns were found in Harbor Porpoises (Crossman et al., 2014), where genetic differentiation depended on hybridization across distinct species. We therefore suggest that genetic differentiation between populations of Copper Rockfish reported in past studies (Buonaccorsi et al., 2002; Seeb, 1998) may have been inflated due to hybridization with Quillback Rockfish.

Nevertheless, the spatial distribution of hybrids suggested limited dispersal of individuals, particularly out of south Puget Sound. The majority of Copper admixed individuals (68%) were found in south Puget Sound, with lower incidence of admixed individuals in north Puget Sound (20%), the Washington Coast (10%), and British Columbia (2%). Additionally, the lack of pure Copper Rockfish in south Puget Sound suggests that this dispersal is highly directional from south Puget Sound into the Salish Sea. This limited directional dispersal from Puget Sound to the outer Washington Coast not only matches similar patterns seen in this study for Quillback Rockfish but has also been found in Yelloweye Rockfish (Andrews et al., 2018) and Pacific Cod (Drinan et al., 2018). These results suggest that all three species in this study have limited directional dispersal out of south Puget Sound.

### 4.3 Implications for fisheries management

Current Puget Sound management policy recognizes three DPSs for Brown, Copper, and Quillback rockfish. All three species are divided into a (1) Puget Sound proper DPS (south of Admiralty Inlet, here SPS), (2) north Puget Sound DPS (San Juan Islands, here NPS), and (3) coastal DPS, which includes populations from California to Alaska (Stout et al., 2001, here WC). Results from our study suggest that the current DPS boundaries do not reflect actual population structure and should therefore be re‐configured. In addition, our genetic results demonstrate that southern Puget Sound populations are isolated from the coast and are unlikely to benefit significantly from the population recovery along the Washington Coast (McQuaw et al., 2021).

We did, however, detect evidence for dispersal and possible gene flow from Puget Sound to the outer coast. In a time of unprecedented environmental change, introgressive hybridization can provide essential genetic variation allowing adaptation to climate change (Arnold et al., 2008; Brauer et al., 2023; Hamilton & Miller, 2016). Quillback Rockfish alleles introgressed into the coastal Copper Rockfish population may allow for more rapid adaptation in the large coastal population. As global climate change rapidly influences the marine environment along the Washington Coast (Miller et al., 2013), hybridization and connectivity processes in Puget Sound may play an important role in the persistence of rockfish species in the region. The results of this study therefore not only confirm the genetic distinctness of Brown, Copper, and Quillback rockfish in Puget Sound but also demonstrate their potential evolutionary significance for each species. As federal DPS designation relies on populations being identified as discrete (i.e., substantially isolated) and significant (i.e., important for the persistence of the species) (Waples, 1995), results from this study suggest that Brown, Copper, and Quillback rockfish may fit these criteria.

## CONFLICT OF INTEREST STATEMENT

The authors declare no conflict of interest.

## Supporting information


Appendix S1.

## Data Availability

Data for this study are available on Dryad (DOI: 10.5061/dryad.j0zpc86mv). This repository contains the raw fastq files, end product files (in VCF format), and relevant metadata.
