# Supplementary material for: Contrasting effect of hybridization on genetic differentiation in three rockfish species with similar life history
Source: Evol Appl. 2024 Jul 19;17(7):e13749. doi: 10.1111/eva.13749 (PMC11259572; doi:10.1111/eva.13749)
Supplement: Supplementary file 1 — Appendix S1. [file EVA-17-e13749-s001.docx]

SUPPLEMENTAL FIGURES


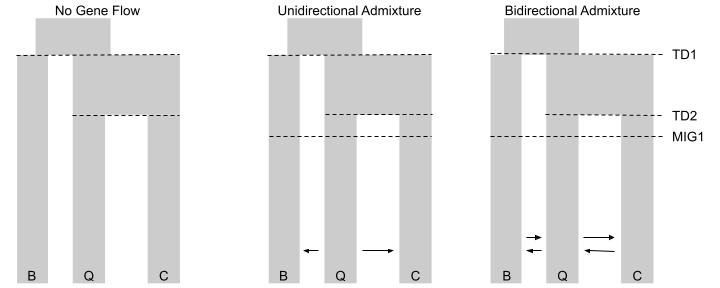


**Supplemental Figure 1. Models simulated in *fastsimcoal*.** Models with different admixture scenarios were compared, including (from left to right) no gene flow after speciation, unidirectional admixture (i.e., admixture from quillback (Q) into brown (B) and copper (C) but not vice versa), and bidirectional admixture (i.e., admixture between quillback and brown and quillback and copper). Mutation rate and generation time were taken from Kolora et al. (2021). The divergence time for the three species was estimated from Hyde & Vetter (2007) and is labeled as TD1 and TD2. MIG1: time where migration between the three species began.


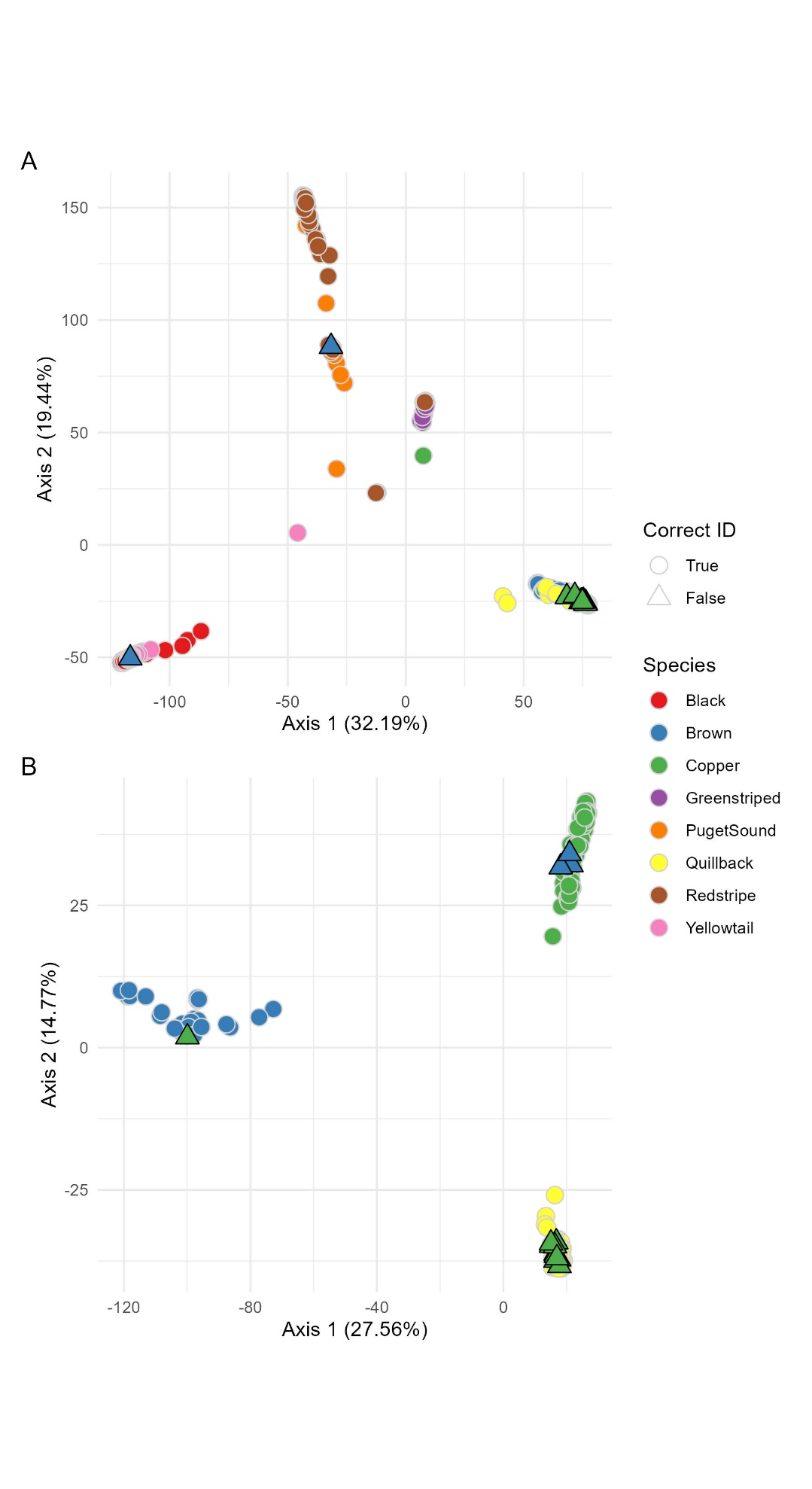


**Supplemental Figure 2. Principal components analysis of eight species of rockfish suggests misidentified individuals.** Each circle represents an individual fish, colored according to field species identification. Each triangle represents an individual where field and genetic identification disagreed. Individuals not included in this study (not Brown, Copper, or Quillback) were collected, sequenced, and filtered using identical protocols (in review). Using the same dataset two PCAs are shown to include (A) all eight species and (B) the species in the current study.

**
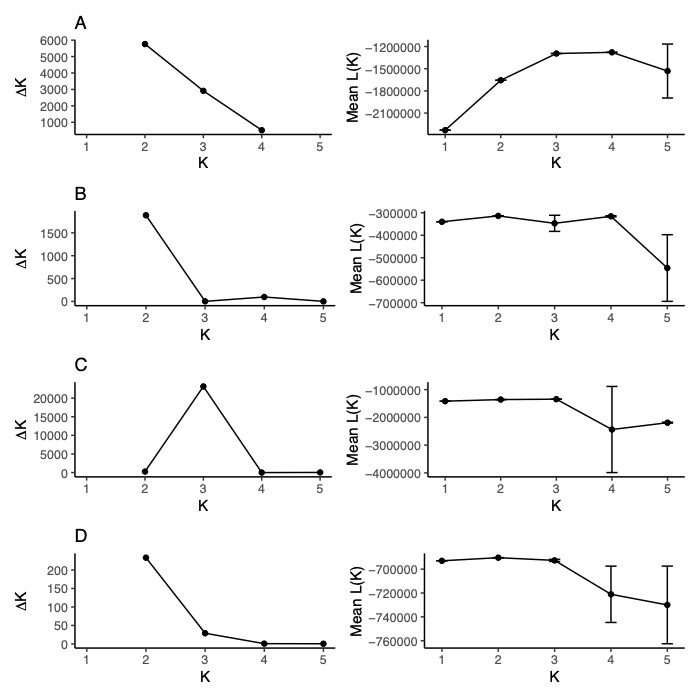
**

**Supplemental Figure 3. Mean likelihood (right, ± 1 SD) and ΔK (left) for *STRUCTURE* replicate runs for interspecific analysis (A) between all three species, and the intraspecific analyses for (B) Brown, (C) Copper, and (D) Quillback rockfish.**

**
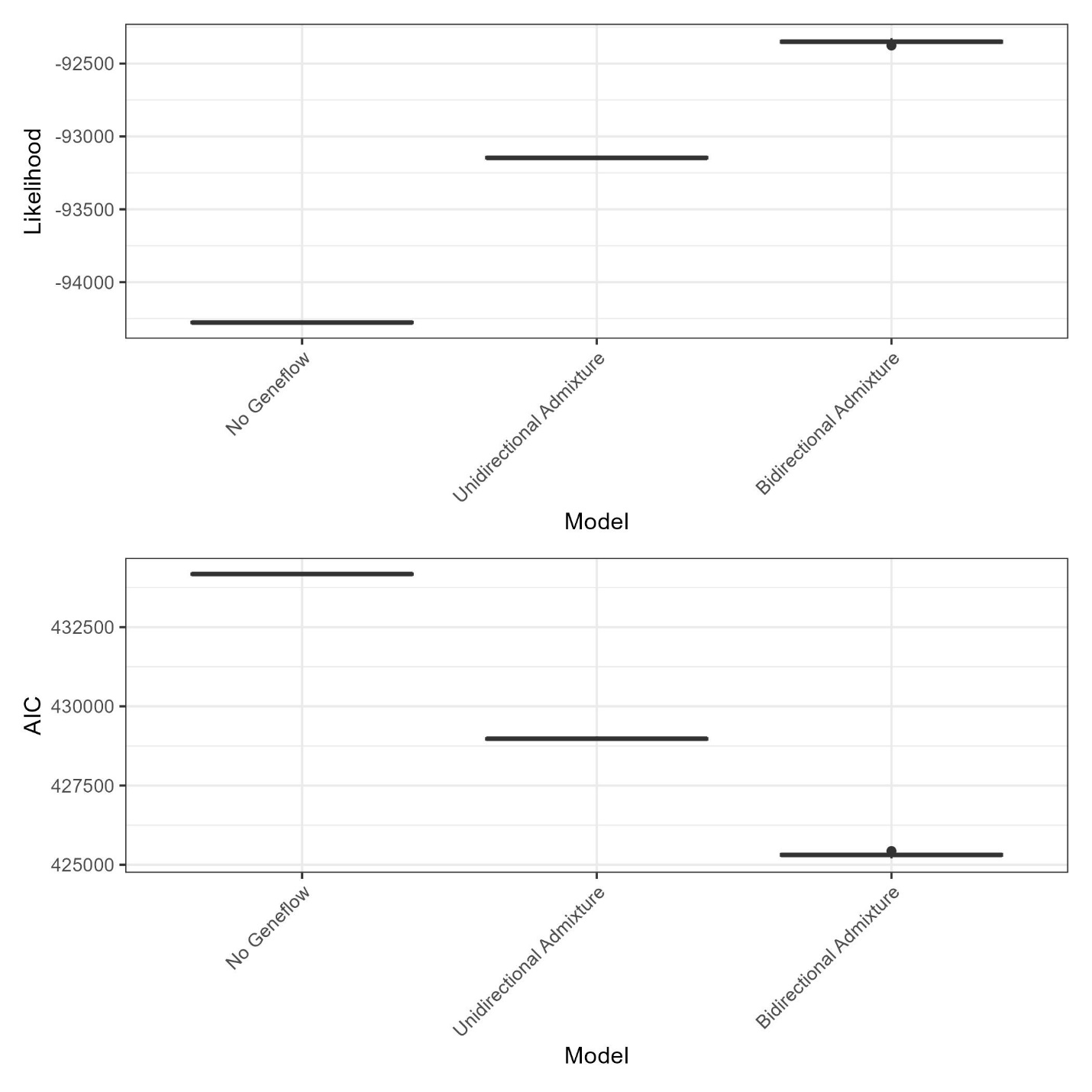
**

**Supplemental Figure 4. Comparison of three demographic models using fastsimcoal.** Box plots show likelihood (top) and Akaike Information Criterion (AIC, bottom) distributions. Models are named based on the degree of admixture allowed in the model after the divergence of the three species. The distribution of the best likelihood and AIC values were calculated by running the model with the best parameter values 100 times.


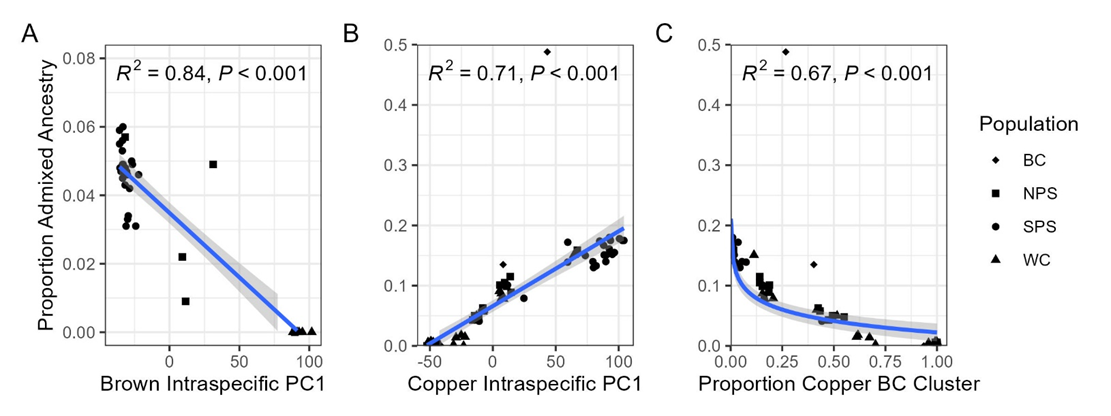


**Supplemental Figure 5. Regression between inter- and intraspecific analyses of (A) Brown and (B&C) Copper Rockfish.** (A) Brown Intraspecific PC1 was taken from PCA of the intraspecific Brown Rockfish dataset. For the Brown plot, the x and y axis come from two separate datasets with 73% different loci. (B) Copper Intraspecific PC1 was taken from PCA of the intraspecific Copper Rockfish dataset. (C) Percent BC cluster was defined as the percent ancestry originating from the ‘green’ BC group (see Fig. 5B) from the *STRUCTURE* results of the intraspecific Copper Rockfish dataset. For B&C plots, the x and y axis come from two separate datasets with 83% different loci. Each dot represents an individual. Percent admixed ancestry was defined as the percent Quillback ancestry from the *STRUCTURE* results of the interspecific Brown/Copper/Quillback dataset (see Fig. 2B).

**
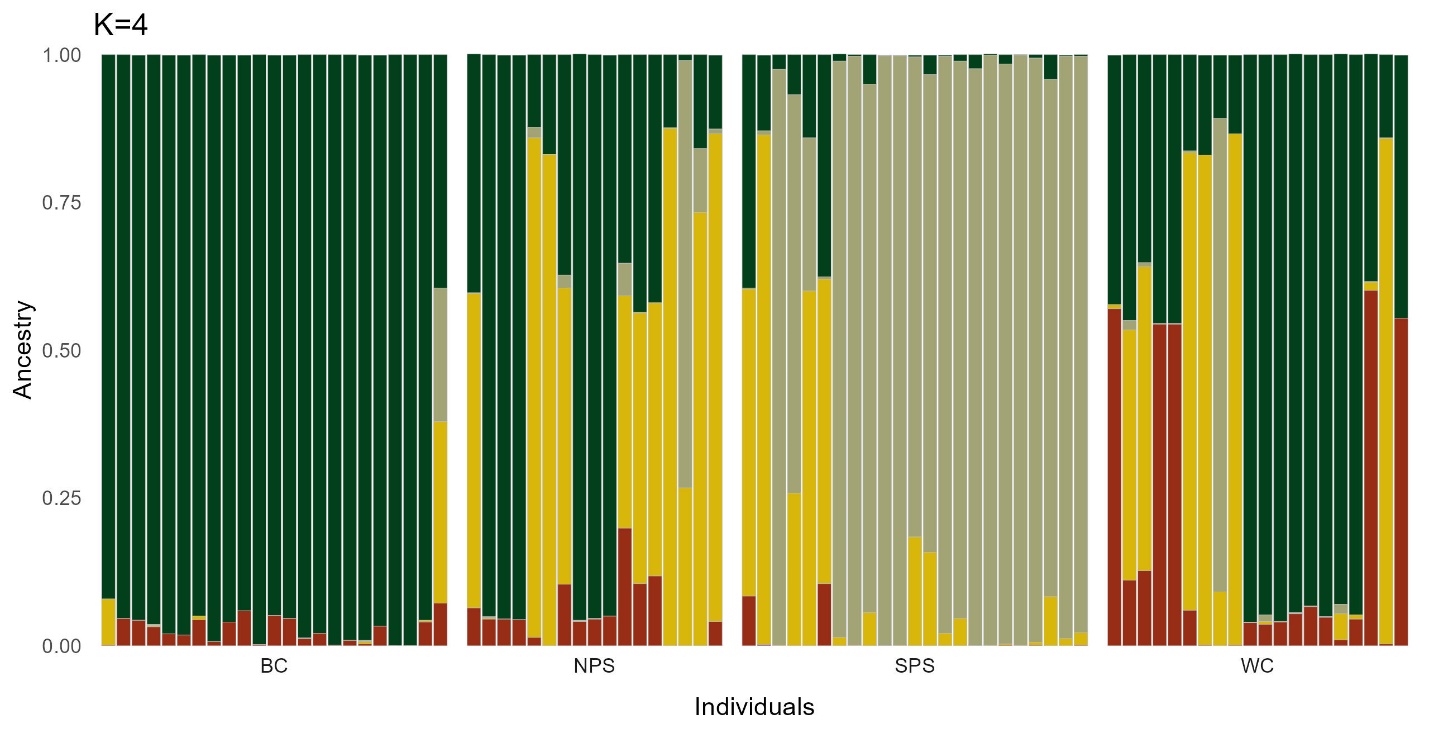
**

**Supplemental Figure 6. *STRUCTURE* plot for K = 4 of Copper Rockfish interspecific dataset.** Each bar represents an individual, and each color the proportion of the genome assigned to each genetic group. Individuals within each location are ordered south to north.


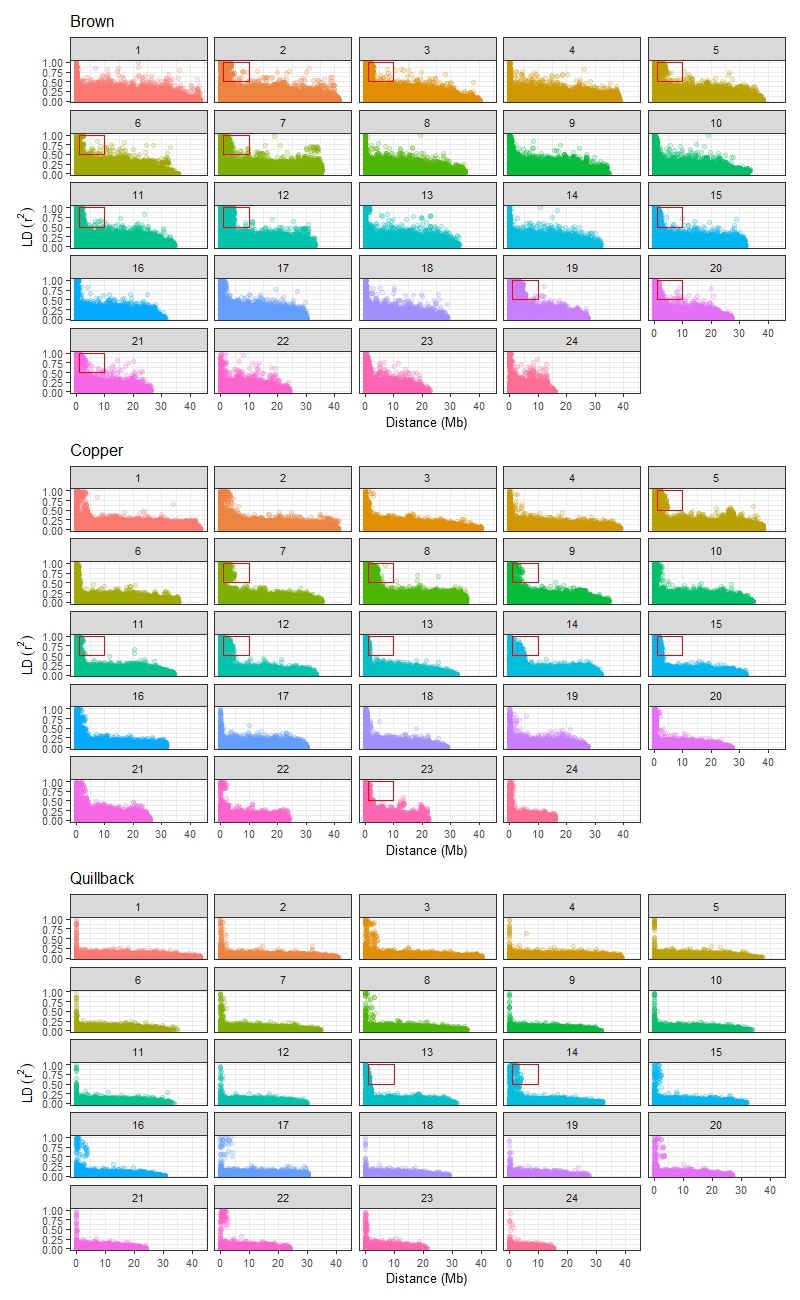


**Supplemental Figure 7. Linkage decay plots for three species of rockfish.** *R*^2^ values between pairs of SNPs were calculated using PLINK v1.07 and plotted against their physical distance on the Honeycomb Rockfish genome. *R*^2^ estimates are typically higher with smaller sample sizes, as seen in our Brown Rockfish dataset. Chromosomes with loci in strong LD (*r*^2^ > 0.5) over extended blocks (distance greater than 1 Mb) are highlighted in red.


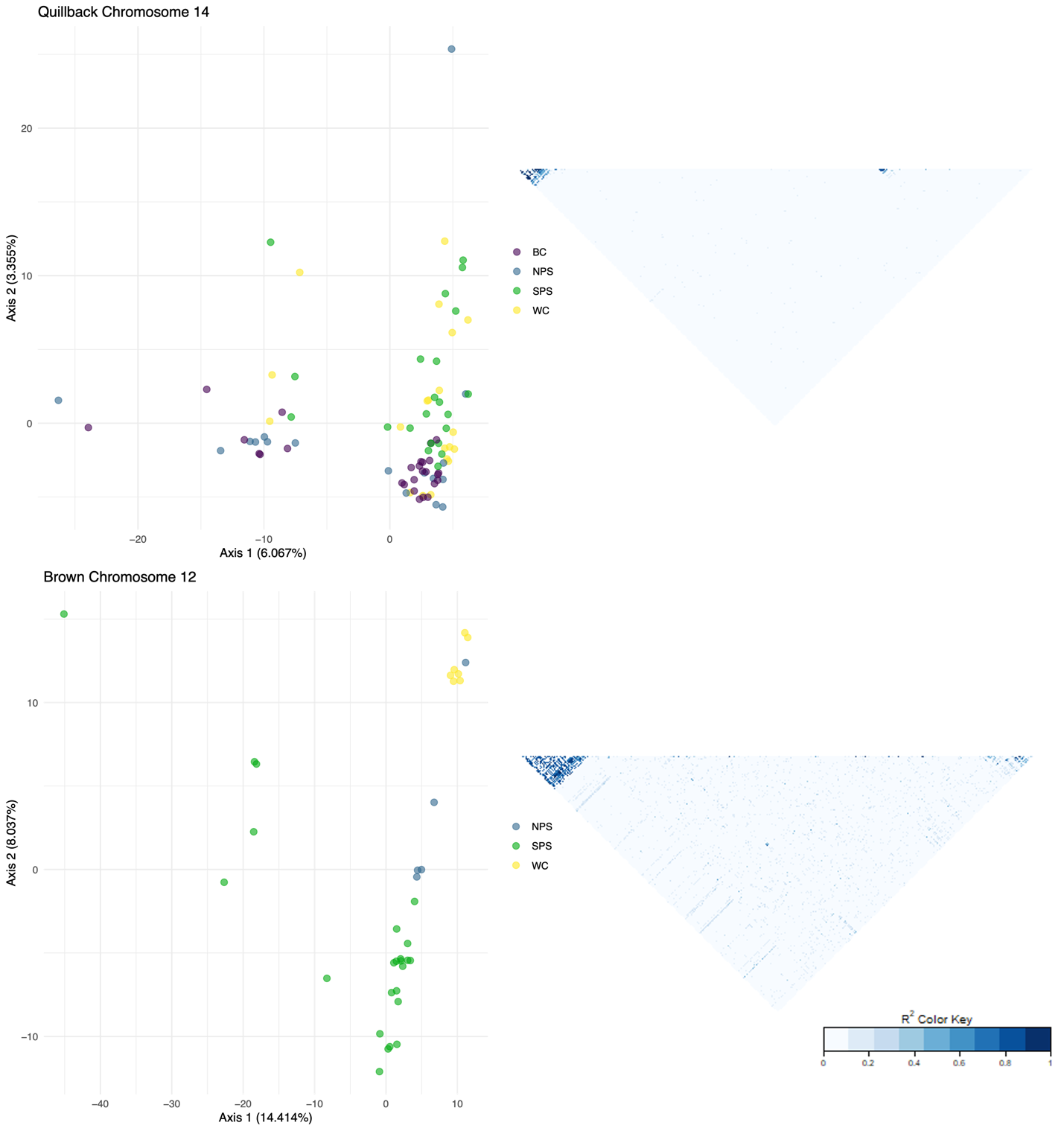


**Supplemental Figure 8. PCA (left) and linkage disequilibrium heatmap (right) for putatively inverted chromosome segments.** A high LD block for Brown Rockfish was identified on chromosome 12 (top). A high LD block for Quillback Rockfish was identified on chromosome 14 (bottom).

SUPPLEMENTAL TABLES

**Supplemental Table 1. Number of Brown, Copper, and Quillback rockfish sampled in this study and Schwenke et al., (2018).** All individuals were identified as non-hybrid in Schwenke et al., (2018) using 5 loci. See Figure 1 for map of sampling regions.

|  | WC | NPS | SPS |
| --- | --- | --- | --- |
| Brown | 13 |  | 13 |
| Copper |  | 5 | 3 |
| Quillback |  | 6 | 22 |

**Supplemental Table 2. Pairwise *F*_ST_ values between three species of rockfish.** Values were estimated with 1,000 bootstraps using the R package *hierfstat* v0.5-11 (Goudet, 2005)

|  | Quillback | Copper |
| --- | --- | --- |
| Copper | 0.417 |  |
| Brown | 0.655 | 0.677 |
